# Extensive endovascular thoracoabdominal aortic septotomy for treatment of malperfusion in acute aortic dissection

**DOI:** 10.1016/j.jvscit.2026.102329

**Published:** 2026-05-22

**Authors:** Kevin C. Eddinger, Lucia Calthorpe, Jade S. Hiramoto

**Affiliations:** a Heart and Vascular Institute, Lehigh Valley Health Network, Allentown, PA; b Department of Surgery, University of California, San Francisco, San Francisco, CA

**Keywords:** Aortic dissection, Malperfusion, Septal fenestration, Cold wire, Septotomy

## Abstract

Management of malperfusion in the setting of acute aortic dissection presents a challenging clinical scenario. We describe a case series of three patients with acute aortic dissection and malperfusion managed with extensive thoracoabdominal endovascular septal fenestration. In all patients, extensive aortic septotomy successfully addressed malperfusion in the acute setting, with evidence of favorable aortic remodeling at follow-up. This technique represents a viable option for the rapid management of malperfusion in the setting of aortic dissection, particularly for patients with anatomy that precludes treatment with thoracic aortic stent-graft repair.

Malperfusion in acute aortic dissection (AAD) is associated with high mortality. Treatment strategies are based on the location of the proximal entry tear (PET) and the extent of aortic involvement, with an emphasis on sealing the PET and/or balancing pressures between the true lumen (TL) and false lumen (FL). In patients with Type A aortic dissection (TAAD), surgical repair of the ascending aorta may be sufficient to relieve distal malperfusion.^1^ However, in patients with persistent malperfusion following TAAD repair, or Type B aortic dissection (TBAD) with malperfusion, descending thoracic aortic interventions may be necessary. Thoracic endovascular aortic repair (TEVAR) for PET coverage is frequently effective in resolving malperfusion and has become the first-line therapy for complicated TBAD.^2^, ^3^, ^4^ However, not all patients are anatomical candidates for TEVAR, and TEVAR in the setting of AAD carries risks of spinal cord ischemia and retrograde TAAD.^5^

We present three cases of extensive aortic septotomy in patients with AAD and visceral malperfusion. All patients had anatomy unsuitable for TEVAR. The patients consented to publication of their case details and images.

## Case 1

**Fig 1:**
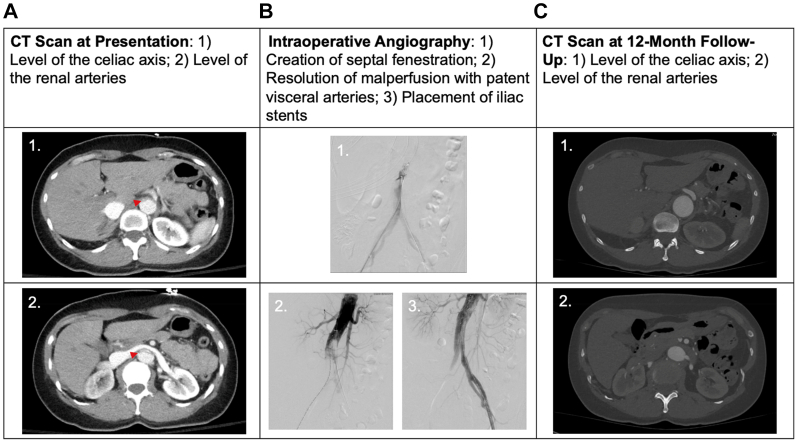
**(A-C)** Case 1. A 44-year-old woman with Marfan syndrome and Society for Vascular Surgery/Society of Thoracic Surgeons (SVS/STS) type A_10B_. *Red arrow*, true lumen (TL). *CT*, Computed tomography.

A 44-year-old woman with Marfan syndrome presented with epigastric and back pain. Computed tomography angiography (CTA) demonstrated TAAD extending from an aneurysmal aortic root to the bilateral common iliac arteries (Society for Vascular Surgery/Society of Thoracic Surgeons [SVS/STS] Type A_10B_) with significant compression of the aortic TL in the visceral segment (Fig 1, *A*). After emergent central aortic repair, her pressor requirement and lactate increased, prompting concern for ongoing visceral malperfusion.

The patient's enlarged arch precluded TEVAR due to an inadequate proximal landing zone. Therefore, bilateral percutaneous common femoral arterial access was obtained. Intravascular ultrasound examination confirmed substantial TL compression throughout the thoracoabdominal aorta. A snare was used to obtain femoral through-and-through Glidewire (Terumo Aortic) access across an existing fenestration in the descending thoracic aorta. Seventy-centimeter sheaths were placed bilaterally for support. The wire was tensioned, and the aortic septum was fenestrated to the aortic bifurcation by pulling on the sheath/through-wire complex bilaterally (Fig 1, *B*).

Subsequent angiography demonstrated a widely patent paravisceral aorta. Septal debris at the aortic bifurcation was treated with an aortic cuff (W. L. Gore & Associates) and kissing iliac balloon-expandable stents (Viabahn VBX, W. L. Gore & Associates). Follow-up imaging at 6 months demonstrated aneurysmal degeneration of the proximal nonfenestrated thoracic aorta, for which she underwent open repair. The fenestrated thoracoabdominal aorta demonstrated favorable remodeling at 12 months with no evidence of prior dissection in these segments (Fig 1, *C*).

## Case 2

A 29-year-old man with Loeys-Dietz syndrome and prior TAAD presented with back pain. CTA demonstrated acute TBAD with entry tears in zones 2 and 3, and extension to the aortic bifurcation (SVS/STS type B_2,9_), with moderate TL compression in the descending thoracic aorta. His left vertebral artery arose directly from the aortic arch.

**Fig 2:**
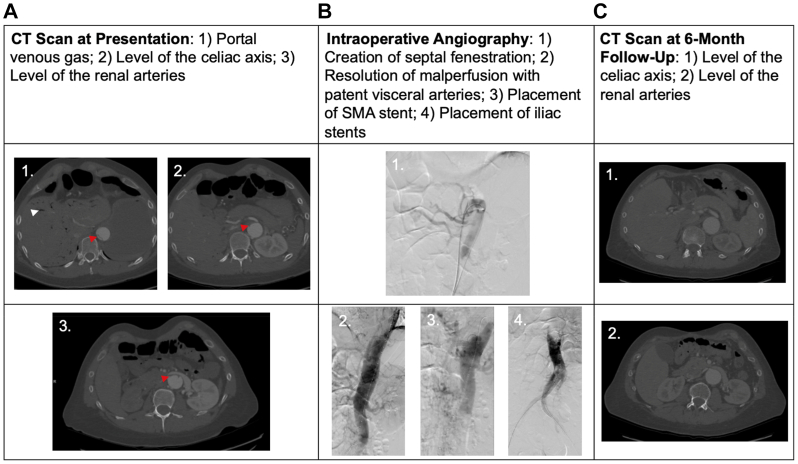
**(A-C)** Case 2. A 29-year-old man with Loeys-Dietz syndrome, prior type A aortic dissection (TAAD), and Society for Vascular Surgery/Society of Thoracic Surgeons (SVS/STS) type B_2,9_. *White arrow*, portal venous gas; *red arrow*, true lumen (TL). *CT*, Computed tomography; *SMA*, superior mesenteric artery.

His worsening clinical condition prompted repeat CTA, which showed complete collapse of the TL with pneumatosis intestinalis and portal venous gas (Fig 2, *A*). Given the PET location, variant arch anatomy, arch dilation (43 mm at the left subclavian artery), and his connective tissue disease, TEVAR was deemed inappropriate. The need for concurrent exploratory laparotomy/bowel resection/open abdomen made open aortic repair high-risk. He underwent septal fenestration from the PET to the aortic bifurcation with the technique described above, with resolution of malperfusion (Fig 2, *B*). A superior mesenteric artery stent was placed due to concern for static obstruction. Intimal debris at the aortic bifurcation required placement of iliac stents. Six months postoperatively, CTA demonstrated favorable remodeling of the descending thoracic and paravisceral aorta, with no evidence of prior dissection seen in these segments, and patent superior mesenteric artery and iliac artery stents (Fig 2, *C*). Residual aneurysmal degeneration of the dissected aortic arch was ultimately treated with open repair.

## Case 3

**Fig 3:**
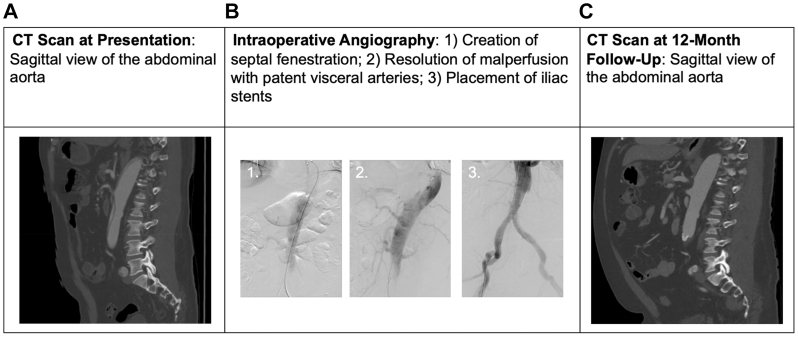
**(A-C)** Case 3. A 44-year-old man with hypertension and Society for Vascular Surgery/Society of Thoracic Surgeons (SVS/STS) type B_2,11L_. *CT*, Computed tomography.

A 44-year-old man with hypertension presented with chest pain and was found to have acute TBAD (SVS/STS type B_2,11L_, PET in zone 3), extending into the left subclavian artery. After initial medical management, he developed abdominal pain and kidney injury. Repeat imaging demonstrated near obliteration of the aortic TL in the visceral segment (Fig 3, *A*).

**Fig 4:**
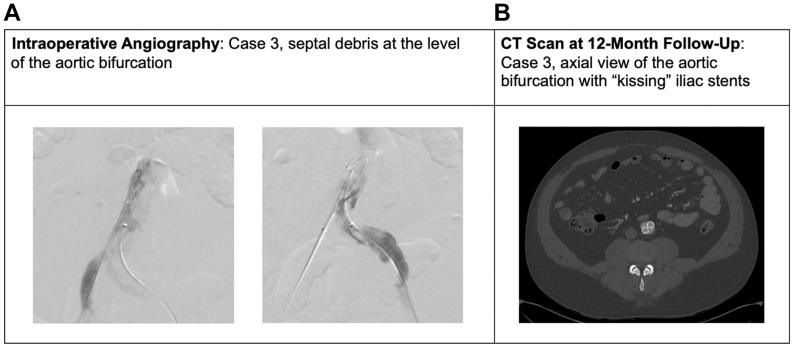
**(A, B)** Case 3. A 44-year-old man with hypertension and Society for Vascular Surgery/Society of Thoracic Surgeons (SVS/STS) type B_2,11L_, septal debris at the aortic bifurcation and kissing iliac stents. *CT*, Computed tomography.

Septotomy was planned, given the unfavorable arch anatomy and involvement of the left subclavian artery. In this case, a Pioneer Plus re-entry device (Philips Healthcare) was used to create a septal fenestration from the TL into the FL of the descending thoracic aorta. Septal fenestration was then performed to the aortic bifurcation, after which there was brisk filling of the mesenteric/renal arteries (Fig 3, *B*). Septal debris at the aortic bifurcation was again managed with kissing stents. Twelve-month follow-up imaging demonstrated a stable residual TBAD in zones 2 through 5 and a single aortic lumen throughout the visceral and infrarenal segments, with patency of the visceral vessels and iliac stents (Figs 3, *C*, and Figs 4, *A*, *B*).

## Discussion

In this series, we describe three patients with AAD and visceral malperfusion managed with extensive thoracoabdominal aortic septal fenestration. In all cases, septotomy relieved malperfusion and showed favorable aortic remodeling on follow-up imaging. This technique merits consideration for the acute management of malperfusion, particularly in settings inappropriate for TEVAR (ie, inadequate proximal landing zone).

The largest series of endovascular fenestration for malperfusion has been described in patients with acute TAAD, followed by staged central aortic repair.^6^ Although this approach has favorable short-term outcomes, partial fenestration of only the supraceliac aortic septum is associated with increased growth rates of both the descending thoracic and abdominal aorta over time.^7^ The technique described in this series may address these limitations by extending the septotomy through the thoracoabdominal aorta and unifying the TL and FL, promoting favorable remodeling while creating a single aortic lumen, which could help to facilitate future procedures, if indicated.

The existing literature describes septotomy for chronic dissection, with radiofrequency energy delivered through the wire, primarily for creating a distal landing zone in chronic TBAD, rescue of stent grafts deployed in the FL, or transeptal stenting of visceral vessels in the setting of fenestrated/branched EVAR.^8^, ^9^, ^10^, ^11^, ^12^, ^13^ However, the literature is sparse regarding septotomy for AAD.

There are several technical considerations when performing extensive septotomy. First, intravascular ultrasound examination is crucial to ensure correct positioning within the TL and FL. Second, we recommend establishing bilateral TL access with buddy wires, in addition to the through-and-through septotomy wire, as regaining aortic TL access after septotomy may be challenging due to septal debris. Given the favorable remodeling and lack of aneurysmal degeneration in the treated portions of the aorta, starting the septotomy as proximally as deemed safe is recommended. However, because these cases were performed within the last 2 years, ongoing surveillance is needed to assess longer-term remodeling outcomes. All patients were managed with septotomy without heat, as it was not necessary, given the septum's friability in the acute setting (the authors have never ruptured the aorta using this technique). Finally, all patients in this series had substantial intimal debris after septotomy, and the need for bilateral iliac stents should be anticipated. If the debris extends significantly above the aortic bifurcation, additional placement of an aortic cuff may be indicated to better accommodate future interventions.

## Conclusions

Extensive aortic septal fenestration appears to be a viable technique for the treatment of malperfusion in AAD, with the advantage of favorable short-term aortic remodeling. This technique may be particularly relevant for patients with connective tissue diseases or those who are not anatomic candidates for TEVAR.

## Funding

None.

## Disclosures

None.
